# Compositional Control of Electrodeposited Co-Ni-Cu Thin Films and Their Behavior in Nitrate Reduction

**DOI:** 10.3390/ma19143122

**Published:** 2026-07-21

**Authors:** Isabella Filagrossi, Md. Bakiul Bashar Rony, Elizabeth J. Podlaha

**Affiliations:** 1 Department of Chemical and Biomolecular Engineering, Clarkson University, Potsdam, NY 13699, USA; isabella.filagrossi@stonybrook.edu (I.F.); ronym@clarkson.edu (M.B.B.R.); 2 Stony Brook University, Stony Brook, NY 11794, USA

**Keywords:** alloy, electrodeposition, nitrate reduction, electrolysis

## Abstract

**Highlights:**

- Anomalous codeposition of Co-Ni-Cu was observed, with Co electrodeposition favored by a tenfold advantage.
- Electrodeposited Co-Ni-Cu thin films were effective in promoting nitrate reduction.
- The nitrogen and ammonia products resulting from nitrate electrolysis were sensitive to the composition of the Co-Ni-Cu cathode.

**Abstract:**

Cobalt–nickel–copper alloys were electrodeposited over a range of current density and with three different aqueous electrolytes having variable metal ion ratios, in order to examine changes in the deposit composition and to use them as cathodes for nitrate electrolysis. The alloys were electrodeposited galvanostatically from a citrate electrolyte onto rotating cylindrical steel substrates. The electrodeposition process exhibited anomalous codeposition behavior, favoring Co reduction over Ni and Cu. These electrodeposits were then used to examine their ability to reduce nitrate in simulated wastewater with 50 mg-N/L of NO_3_^−^, sodium chloride, and sodium sulfate. Nitrate conversion and selectivity were characterized after electrolysis in a single-compartment cell with the alloys serving as the working electrode. Despite co-evolving hydrogen, the electrodeposited alloys were effective at generating both N_2_ at high electrolysis current densities and ammonia species at lower values, with the deposit composition also affecting the conversion and products. It is the first demonstration of using Co-Ni-Cu ternary alloys for nitrate reduction.

## 1. Introduction

The fabrication of Co-Ni-Cu thin films by electrodeposition is a cost-effective strategy and is used to readily coat conductive substrates of irregular shape and size. However, the reduction in a combination of these metal ions to form a ternary alloy is not straightforward. In the literature, the ternary Co-Ni-Cu alloys have been electrodeposited for different applications, principally for their magnetic [1,2] and mechanical behavior [3]. Gómez et al. [1] examined the nature of the electrodeposited process of Co-Ni-Cu alloys in citrate electrolytes over a range of Co(II)-rich electrolyte concentrations with the aim of obtaining Co-rich alloys. They observed an anomalous codeposition behavior, where the least noble ion reduction reaction, in this case, Co, deposited in a larger amount than anticipated from its electrolyte concentration. This observation would be expected from the known anomalous codeposition behavior, although the extent of enhancement is hard to predict. Anomalous codeposition has been recognized in the iron-group metal ions of Fe, Co, and Ni, where codeposition favors Fe > Co > Ni, despite the order of nobility being the opposite, Ni > Co > Fe [4], with examples in both binary [5,6] and ternary systems [7,8]. Binary combinations of Co, Ni, and Fe alloys have been reported to be effective in reducing nitrate, such as Cu-Ni [9] and Cu-Co [10]. Hence, the objective of this study is twofold: i. to examine conditions to electrodeposit a ternary Co-Ni-Cu alloy thin film with control of the deposit composition; and ii. to characterize nitrate reduction using this ternary alloy. To mimic a wastewater environment, sodium chloride was present in a near-neutral-pH electrolyte.

Since nitrate in wastewater is often not concentrated, its limiting current density is low and consequently, a significant amount of hydrogen would also co-evolve due to the hydrogen evolution reaction (HER), lowering the Faradaic efficiency (FE). The reduction of nitrate is more noble than HER; thus, in theory, 100% Faradaic efficiency (FE) is plausible, but due to the nitrate reduction reaction’s sluggish kinetics, a larger overpotential is often required so that the potential enters the HER regime. Recently, Zhou et al. [11] have shown that a PdCuCo medium-entropy alloy fabricated by a solvothermal process reduced nitrate to ammonia at 99.7% FE; a similarly high Faradaic efficiency for a Ru-Cu onto a Ni foam material was achieved [12]. One motivation to investigate transition-metal alloys, without the addition of Pd or Ru, is to reduce cost. Also, HER can provide a supply of reactive adsorbed H(I)_ads_ species that is known to accelerate nitrate reduction [13]. An additional advantage of gas evolution is that the bubbles can provide mixing near the electrode surface, which is particularly advantageous if the concentration of nitrate is low, creating a low limiting current density and hence reaction rate. The micro-convection increases this mass-transport-limited rate. Hydrogen itself is also a value-added product. In this study, nitrate reduction occurs with the reduction of water, by design, with expected low Faradaic efficiency in order to evaluate if the Co-Ni-Cu electrodeposited ternary alloys can promote nitrate reduction in a wastewater environment.

Nitrate (${NO}_{3}^{-}$) is a nutrient, but if its concentration in water is too high, greater than 10 mg-N/L, according to the US EPA [14], then it is a pollutant, resulting in nutrient overload in waterways that can lead to eutrophication and harmful algae blooms [15,16], as well as numerous health issues in humans when ingested [17,18,19,20]. Some methods of remediation are separation processes that require post-treatment, such as ion exchange [21] and reverse osmosis [22], unlike electrochemical reduction, which has the advantage of directly destroying the nitrate to products such as nitrogen or ammonia [23,24],

(1) $${2NO}_{3}^{-}+10e^{-}+6H_{2}O\to N_{2}+12OH^{-}$$

(2) $${NO}_{3}^{-}+8e^{-}+6H_{2}O\to {NH}_{3}+9OH^{-}$$

Noble metals (e.g., Pt [25,26,27], Pd [28]) are well-recognized electrocatalysts to reduce nitrate on account of their excellent electrical conductivity and strong adsorption capacity for reaction intermediates. However, these materials are also very good hydrogen evolution reaction (HER) electrocatalysts and consequently compete with the nitrate reduction reaction, lowering the efficiency for nitrate reduction and, in the extreme case, can inhibit nitrate reduction by blocking adsorption sites. The high cost of noble metals also limits their commercial acceptance. In contrast, copper has lower HER kinetics and has been widely studied as an electrocatalyst due to its comparatively high corrosion resistance relative to other non-noble metals, in both acidic conditions [29,30] and in weakly alkaline environments [31], although it favors ammonia over nitrogen products [32], and forms less active oxides at high pH. Additionally, the active sites of copper electrocatalysts have been observed to be poisoned by certain anions, such as the chloride ion, Cl^−^ [33]. In contrast, Cl^−^ has been reported to promote the selectivity of N_2_ over ammonia on nickel substrates [34]. Fajardo et al. [35] found that nickel, carbon, and tin electrodes had a higher N_2_ selectivity compared to Co and Fe.

It has been recognized that the presence of copper in copper alloys helps to facilitate the nitrate reduction rate-determining step to a nitrite, NO_2_^−^, intermediate. Other metal elements can affect the copper electronic d-band center and surface potential, which changes the adsorption of reactants and intermediates to promote the subsequent reactions to ammonia or nitrogen [36,37,38,39]. A range of Cu-based alloys have been examined, offering more flexibility in product selectivity; these include copper–nickel [9], copper–zinc [40], copper–tin [41], iron-coated copper foam [42], copper–rhodium [43], copper–palladium [44], and copper–cobalt [10]. More recently, Chen et al. [45] and Wang et al. [46] examined bimetallic Co/Ni electrocatalysts and demonstrated selectivity toward N_2_. Yan et al. [47] created iron-group (Fe, Co, or Ni) nanosheets onto Cu nanowires to construct Cu-Fe, Cu-Co, and Cu-Ni tandem nano-electrocatalysts, motivated by having the Cu sites facilitate the conversion of ${NO}_{3}^{-}$ to ${NO}_{2}^{-}$, while the Co or Ni sites would enhance the further reduction of ${NO}_{2}^{-}$ to the desired products. Combining Cu with Co resulted in a high conversion to ammonia, while combining Cu and Ni enabled the further reduction of ${NO}_{2}^{-}$ at lower overpotentials, leading to over 99% NH_3_ Faradaic efficiency, albeit at a low current density. Fe sites were not helpful to promote the further reduction of ${NO}_{2}^{-}$.

Here, a novel combination of Co-Ni-Cu electrodeposits is examined in the presence of Cl^−^ with the desire to reduce nitrate more selectively to N_2_ from a near-neutral nitrate electrolyte. The electrodeposition electrolyte is designed to have a smaller amount of copper ions, Cu(II), compared to Co(II) and Ni(II), to create a mass-transport limit of the copper partial current density. A rotating cylinder working electrode was employed to control the steady-state Cu limiting current density. Both the nickel and cobalt ion concentrations were purposely in excess of the copper ion concentration so that their reduction rates were kinetically controlled in order to vary the alloy composition by a change in current density. Following deposition, nitrate reduction is characterized from a low-concentration simulated wastewater electrolyte with 50 mg-N/L of NO_3_^−^ that inevitably will have a competing hydrogen evolution reaction. Unlike literature studies that try to maximize NO_3_^−^ conversion, this study investigates the influence of the alloy composition with a co-evolving side reaction on conversion and product selectivity.

## 2. Materials and Methods

The Co-Ni-Cu alloys were electrodeposited from a citrate electrolyte, containing a total cobalt sulfate and nickel sulfate metal ion concentration of 0.7 M, with 0.008 M copper sulfate hydrate, 0.25 M sodium citrate, 1 g/L sodium lauryl sulfate, and 10 mL 28–30% ammonium hydroxide in deionized and ultra-filtered water (DIUF). All chemicals were purchased from Thermo Fisher Scientific (Waltham, MA, USA). Different ratios of nickel to cobalt ions were investigated, as listed in Table 1. The alloys were electrodeposited onto steel cylindrical substrates (#1018) having a 1.5 cm diameter and a surface area of 3.0 cm^2^. Prior to electrodeposition, the steel substrates were washed using E-SOL foamy cleaner (Cambridge Diagnostics Products, Inc., Fort Lauderdale, FL, USA), polished with SiC sandpaper grit #1000, followed by a polish using 1-micron alumina paste, and then rinsed and treated in an ultrasonic cleaner for 5 min prior to electrodeposition. Galvanostatic deposition and voltammetry were carried out with a galvanostat/potentiostat (Pine Instrument Co., Grove City, PA, USA) in a single-compartment cell with the steel cylinder rotating electrode (RCE) as the working electrode, a titanium-platinum mesh counter electrode, and a saturated Ag/AgCl reference electrode. The counter electrode was concentric to the RCE working electrode, and the reference electrode was placed between the counter electrode and working electrode. The steel RCE was the substrate for electrodeposition of the ternary alloys at different current densities. Deposition occurred when the RCE was rotated at 1000 rpm to maintain a constant hydrodynamic environment. The scan rate during polarization experiments was 5 mV/s, and the potential was corrected for ohmic drop. The ohmic resistance was measured with electrochemical impedance spectroscopy (EIS).

Following the deposition, the composition was analyzed using X-ray fluorescence (XRF) spectroscopy (Omicron, Kevex Co., Scotts Valley, CA, USA) in air at 30 keV, 2 mA, and an acquisition time of 60 s. The morphology was characterized by scanning electron microscopy (JEOL JSM 7900F SEM, Tokyo, Japan).

Nitrate reduction was performed using a solution that contained 0.304 g/L sodium nitrate (50 mg-N/L), 0.5 g/L sodium chloride, and 1 g/L sodium sulfate. The pH was 6.4 and was used without adjustment. It was performed in a single-compartment cell, with the deposited Co-Ni-Cu RCE serving as the cathode, with a platinum coil anode and an Ag/AgCl reference electrode. As a comparison for polarization, an equivalent nitrite electrolyte was used, with 50 mg-N/L of sodium nitrite, 0.5 g/L sodium chloride, and 1 g/L sodium sulfate. The Co-Ni-Cu RCE was rotated at 1000 rpm. Nitrate electrolysis was conducted for 2 h in 0.12 L of electrolyte at variable current density.

Post nitrate reduction, the solution was analyzed for nitrate, nitrite, and ammonia concentrations using the following testing kits: TNT 836/830 ${NO}_{3}^{-}$, TNT 840 ${NO}_{2}^{-}$ and TNT 832 dissolved free ammonia and ammonium ion (Hach, Co., Ames, IA, USA) together with a Hach DR3900 Benchtop Visible Spectrophotometer. The kits are based on a colorimetric reaction having proprietary reagents with labeled barcodes that the spectrophotometer reads and correspond to the wavelength range used in the analysis. Calibration curves of known nitrate, nitrite, and ammonia concentrations in the electrolyte used here were used to validate the results.

## 3. Results

Alloys were electrodeposited at conditions above the copper limiting current density determined through polarization. These deposition conditions were −5, −10, −15, and −20 mA/cm^2^. The subsequent nitrate electrolysis targeted the region where HER occurred, as suggested by an upward inflection of a polarization curve, but also tried to avoid conditions when HER was excessively vigorous, as determined by visible bubble formation. The electrolysis conditions selected were the same as the electrodeposition conditions: −5, −10, −15, and −20 mA/cm^2^.

### 3.1. Electrodeposition

**Figure 1:**
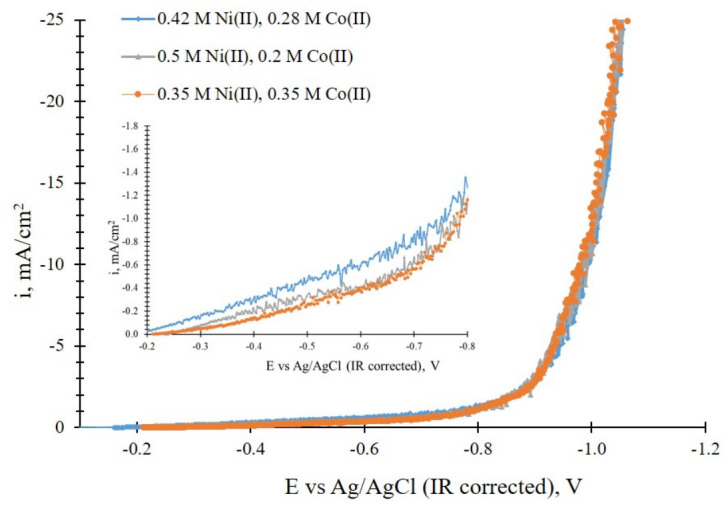
Polarization curve of the electrodeposition electrolytes with different Ni(II) and Co(II) ion concentrations with the total less noble metal ion concentration of 0.7 M, and 0.008 M Cu(II) at 1000 rpm, 5 mV/s. Inset: expanded view at low current density.

The polarization curves for the three different electrodeposition electrolytes are shown in Figure 1. All three electrolytes had the same amount of copper with different ratios of Ni(II) and Co(II). The more noble Cu deposition is expected to be most prominent at more noble potentials, and an inflection point at around −0.75 V indicates the region where Ni and Co commence deposition along with the hydrogen evolution and oxygen reduction side reactions. Therefore, the selected current densities of −5, −10, −15, and −20 mA/cm^2^ were chosen to deposit the ternary alloy.

Despite the overlapping polarization curves, the deposit compositions from the varying electrolyte solutions were different. Table 2, Table 3 and Table 4 summarize the compositions of the electrodeposited alloys. The metal composition was an average of five data points along the electrode length, and the standard deviation was used to capture its variation in the composition as a consequence of a small current distribution. As the current density increased, the amount of cobalt and nickel deposited increased while the amount of copper decreased, confirming the mass-transport control of Cu(II) reduction and the kinetic control of the Ni(II) and Co(II) reduction. The ratio of Co/Ni is nearly independent of the current density. The ratio in the deposit follows a trend based on the electrolyte concentration. When the ratio of metal ions of Co(II)/Ni(II) in the electrolyte was 1, Table 2, from the electrolyte containing the lowest nickel ion concentration (0.35 M), the resulting deposit composition had a Co/Ni wt%/wt% ratio of roughly 10. When the electrolyte ratio was 0.67, Table 3, with a higher nickel ion concentration (0.42 M), a deposit ratio of roughly 7 was obtained. An electrolyte ratio of 0.4, Table 4, with the highest nickel ion concentration examined (0.5 M), resulted in a deposit ratio of 4. In all cases, significantly more Co compared to Ni was found in the resulting deposit compared with the electrolyte, in line with the expected anomalous codeposition. What was not anticipated was the almost consistent tenfold increase in cobalt content in each electrolyte, regardless of the ratio and applied current density.

**Figure 2:**
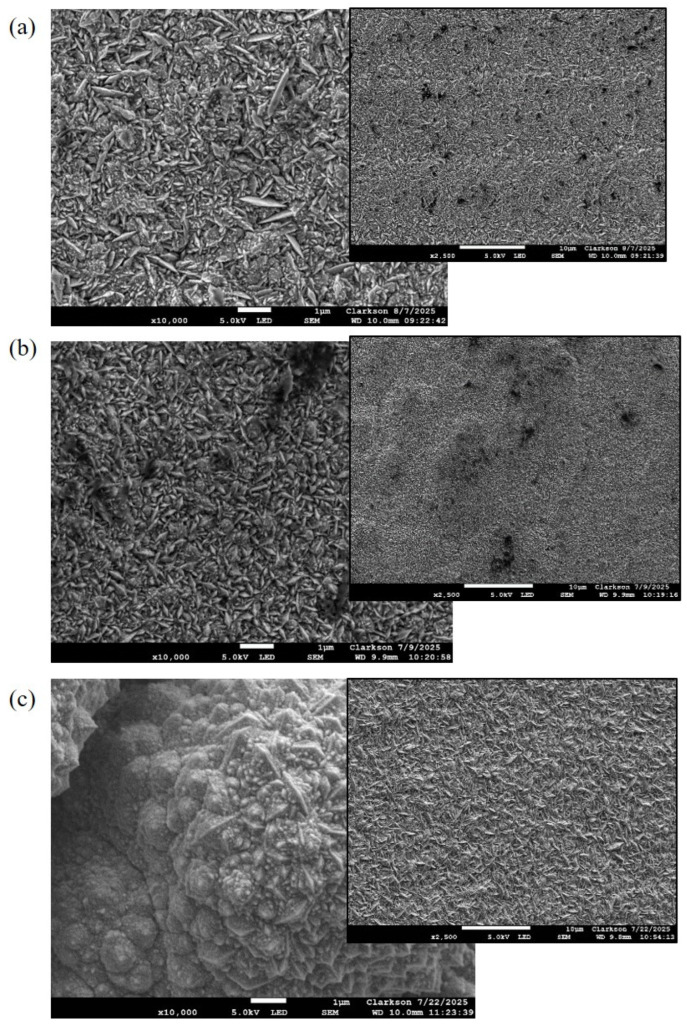
SEM of deposits, (**a**) Co/Ni 10.6 wt%/wt%, (**b**) Co/Ni 7.5 wt%/wt%, (**c**) Co/Ni 4.3 wt%/wt% having similar amounts of Cu (**a**) 13 wt%, (**b**,**c**) 15.8 wt%; magnification was ×10,000; inset magnification was ×2500.

Three representative morphologies of the electrode surface are shown in Figure 2 for the different Co/Ni ratio deposit concentrations having nearly the same Cu content. The Co-rich deposit, Figure 2a, has needle-like and plate morphologies of varying sizes (e.g., 0.1–1.5 μm) at high magnification, similar to those observed by Gómez et al. [1]. At low magnification, there are numerous round pits of micron scale. As the amount of cobalt in the deposit is decreased, the feature size and shape change. Figure 2b, with a Co/Ni deposit wt% ratio of 7.5, shows smaller features at high magnification, and fewer pits at low magnification. Even lower Co content, a Co/Ni deposit wt% ratio of 4 in Figure 2c, results in a deposit that has a combination of nodules and plates at high magnification, and is rougher at low magnification.

### 3.2. Nitrate Electrolysis

**Figure 3:**
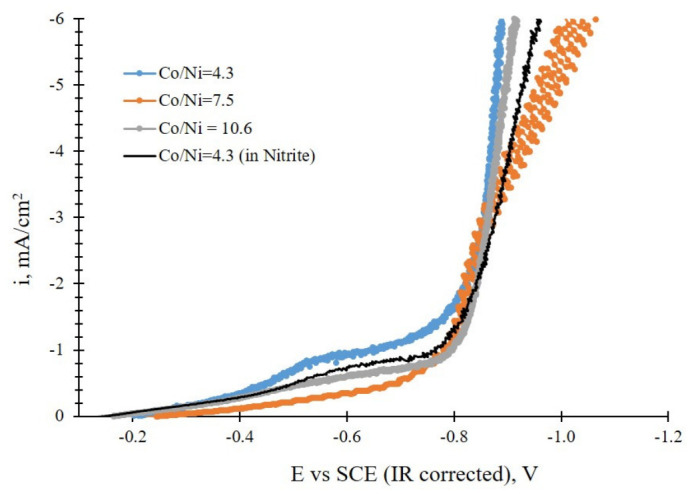
Polarization curves in the nitrate electrolyte with three different Co-Ni-Cu electrodes from the nitrate electrolyte, along with one electrode type generated from a nitrite electrolyte; scan rate was 5 mV/s.

Following electrodeposition, nitrate polarization curves, Figure 3, were obtained from the representative deposit shown in Figure 2 that is electrodeposited from the three different electrolytes and thus having variable Co/Ni deposit ratios but similar amounts of Cu. Figure 3 shows that, in all cases, there is a sharp rise in current density at −0.8 V vs. Ag/AgCl, where the hydrogen evolution reaction and nitrate reduction occur simultaneously. At cathodic current densities near |2| mA/cm^2^ a plateau is observed. Similar plateaus have been observed by others [48,49] that have been attributed to either a limiting current density of the nitrate to $N_{2}$ and/or ${NH}_{3}$, as in Equations (1) and (2), as well as the partial reduction in nitrate to nitrite, ${NO}_{2}^{-}$ according to reaction (3).

(3) $${NO}_{3}^{-}+2e^{-}+H_{2}O\to {NO}_{2}^{-}+2OH^{-}$$

A polarization curve of one Co-Ni-Cu electrode in a nitrite ${NO}_{2}^{-},\;$ electrolyte having the same supporting ions as the nitrate electrolyte is also compared in Figure 3. At a similar potential range, a plateau is observed that would indicate the reduction of ${NO}_{2}^{-},$ to either nitrogen or ammonia.

(4) $$2{NO}_{2}^{-}+6e^{-}+4H_{2}O\to N_{2}+8OH^{-}$$

(5) $${NO}_{2}^{-}+6e^{-}+5H_{2}O\to {NH}_{3}+7OH^{-}$$

The limiting current density in the nitrite solution is lower than the comparable one for nitrate, having the same bulk concentration. Since there are 6 electrons in the reduction of ${NO}_{2}^{-}\;$ to the ammonia product versus 8 for the reduction of ${NO}_{3}^{-}$ to ammonia, or 10 electrons to $N_{2}$, the lower limiting current density would be expected. The ratio of the limiting current density between the nitrate and nitrite reactant is 1.2. If complete conversion of nitrate to ammonia and nitrite to ammonia occurred, the ratio of the limiting current densities should be 8/6 = 1.33, or if complete conversion of nitrate and nitrite to nitrogen occurred, then this ratio would be 10/6 = 1.66. The value of 1.2 obtained indicates that the products forming at current densities close to the nitrate plateau current density region would be dominated by ammonia and possibly partially reduced nitrate to nitrite or nitrogen. To avoid nitrite generation, and to be in a range where $N_{2}$ is favored, the applied current density range was chosen to be above |2| mA/cm^2^.

**Figure 4:**
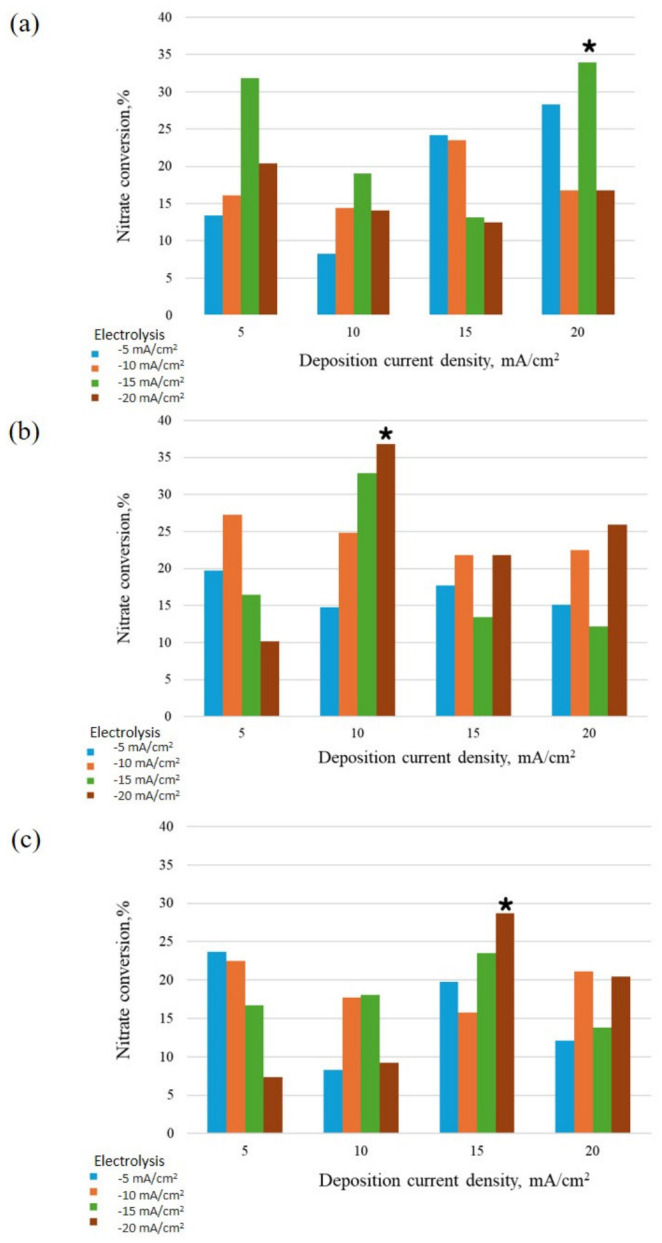
Nitrate conversion for different electrodeposits at varying electrolysis current densities for 2 h. Electrodeposited from the following electrolytes: (**a**) low nickel electrolyte concentration; Ni(II), Co(II), and Cu(II) concentrations are: 0.35 M, 0.35 M, and 0.008 M, respectively. (**b**) Medium nickel electrolyte concentration; Ni(II), Co(II), and Cu(II) concentrations are: 0.42 M, 0.28 M, and 0.008 M, respectively. (**c**) High nickel electrolyte concentration; Ni(II), Co(II), and Cu(II) concentrations are: 0.5 M, 0.2 M, and 0.008 M, respectively. * indicated the highest nitrate conversion achieved for the electrolyte.

Nitrate, nitrite, and ammonia concentrations were determined after nitrate reduction from an electrolyte containing 50 mg-N/L for 2 h at 1000 rpm. Figure 4 shows the nitrate conversion for deposits fabricated using the three electrolytes at different current densities. The colored bars represent the different electrolysis current densities in the nitrate electrolyte. The electrolysis current densities did not seem to have a trend for a given alloy composition. Along the *x*-axis, the deposit current density, from low to high, has the most change in copper concentration. Figure 4a has a higher Co/Ni ratio, ~10; in Figure 4b, the Co/Ni ratio is ~7; and in Figure 4c, the Co/Ni ratio is 4. In each Co-Ni-Cu alloy system, there is a high conversion point indicated with asterisks that occurs at an electrolysis current density of either −15 mA/cm^2^ with the high Co content, Figure 4a, or −20 mA/cm^2^ with the lower Co/Ni ratios, Figure 4b,c.

The composition alone does not predict nitrate activity; it is also influenced by the applied current density. Interestingly, inspecting the ratio of the composition of the Ni + Co with respect to Cu, $\frac{\left(X_{Ni}+X_{Co}\right)}{Cu}$, and normalizing it with its deposition current density, *i*, was observed to correlate with the higher observed nitrate conversion. For the highest nitrate conversion from the different electrolytes in Figure 4, as indicated by the asterisk, they all had a similar value of this ratio, $\frac{\frac{\left(X_{Ni}+X_{Co}\right)}{Cu}}{i}$, of −0.51 to −0.53% cm^2^/%mA. In contrast, if the conversion was considerably lower, the value of the ratio differed; an example is provided in Table 5. In Table 5, the high conversions (*) from each electrolyte: Table 2, Figure 4a, (a), Table 3, Figure 4b, (b), and Table 4, Figure 4c, (c) and one low conversion value of nitrate, Table 2, Figure 4a, are given. The high conversions fit into a common ratio while the low conversion value lies outside that ratio.

**Figure 5:**
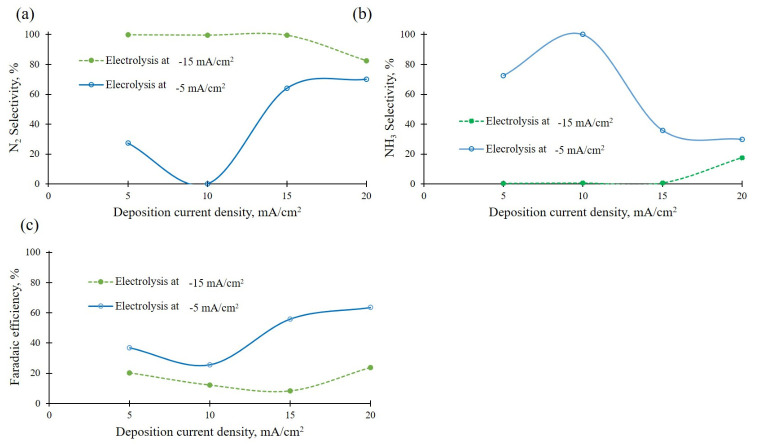
Product analyses from electrolysis of the deposits fabricated from the low nickel electrolyte concentration; Ni(II), Co(II), and Cu(II) concentrations are: 0.35 M, 0.35 M, and 0.008 M, respectively. (**a**) Nitrogen selectivity, (**b**) ammonia selectivity, and (**c**) overall Faradaic efficiency.

The selectivity and FE results for the alloys electrodeposited from the electrolyte containing 0.35 M Ni(II), 0.35 M Co(II) and 0.008 M Cu(II) are shown in Figure 5 for two electrolysis conditions, a low value cathodic current density (−5 mA/cm^2^) and a higher cathodic current density (−15 mA/cm^2^) accompanied with a substantial increase in the HER, and hence an expected low Faradaic efficiency for nitrate reduction. The alloy used for nitrate electrolysis had a Co/Ni ratio of approximately 10 with varying amounts of Cu. The nitrate, nitrite, and ammonia species were measured, and the amount of nitrogen was indirectly determined through a mass balance after 2 h of electrolysis. The products are presented as selectivities defined as follows.

(6) $$S_{N_{2}}=\frac{{\left[N_{2},\frac{mg-N}{L}\right]}_{t=2\;h}}{{\left[{NO}_{3}^{-},\;\frac{mg-N}{L}\right]}_{t=0}-{\left[{NO}_{3}^{-},\;\frac{mg-N}{L}\right]}_{t=2\;h}}\;$$

(7) $$S_{{NH}_{3}}=\frac{{\left[{NH}_{3},\;\frac{mg-N}{L}\right]}_{t=2\;h}}{{\left[{NO}_{3}^{-},\;\frac{mg-N}{L}\right]}_{t=0}-{\left[{NO}_{3}^{-},\;\frac{mg-N}{L}\right]}_{t=2\;h}}\;$$

The Faradaic efficiency, FE, was determined by Faraday’s law, assuming a steady state,

(8) $$FE,\;\%=100\left(\frac{5FC_{N_{2}}V}{Q\left(14.01\times 10^{3}\right)}+\frac{2FC_{NO_{2}^{-}}V}{Q\left(14.01\times 10^{3}\right)}+\frac{8FC_{{NH}_{3}}V}{Q\left(14.01\times 10^{3}\right)}\right)$$

where *V* is the volume, *F* Faraday’s constant, *Q* the charge passed (*I*·7200 As), and with concentrations, *C*, in mg-N/L. Nitrite was not detected in the electrolyte following electrolysis. Note that the total nitrogen of all ammonia species is detected, and it is assumed for simplicity to represent ammonia species as NH_3_.

In Figure 5, as the alloy deposition cathodic current density increases, represented along the *x*-axis, there is less copper in the deposit and an increase in Co and Ni (Table 2) that influences the selectivity and Faradaic efficiency. The resulting products after nitrate electrolysis at the low current density (−5 mA/cm^2^) had a high selectivity for ammonia, using electrodeposits with a high Cu content in the Co-Ni-Cu alloy. However, even at this electrolysis current density, as the Cu content dropped in the deposit, so did the selectivity towards ammonia. In contrast, at a high electrolysis current density (−15 mA/cm^2^), the same high Cu-content Co-Ni-Cu electrodeposit favored N_2_ production with a lower Faradaic efficiency, as expected with an increase in HER. In all cases, the Faradaic efficiency was low, but that was by design in order to probe whether the HER permitted nitrate reduction at all.

## 4. Discussion

The electrodeposition of Co-Ni-Cu was observed to deposit in an anomalous way since Co was preferentially deposited over Ni. The Cu(II) reduction, being more noble, would be the dominant reaction, but it was quenched by operating under mass-transport control due to its low concentration in the electrolyte. Existing mechanisms for anomalous codeposition [6,7] assume that the less noble species reduction reaction, in this case Ni(II) reduction, is inhibited by the more noble Co(I) adsorbed species during the reduction in Co(II). However, this does not explain the interesting constant ratio of Co/Ni in the deposit with current density at a given Co(II)/Ni(II) ratio in the electrolyte. An alternative view, Equations (9) and (10), might be one where both Co(II) and Ni(II) ions reduce together, forming an adsorbed intermediate and then subsequently reduced to a metal in a particular ratio. The copper is decoupled from the Co-Ni mechanism because, as observed here, it is under mass-transfer control, further supported by its content in the alloy decreasing with the applied current density.

(9) $$xCo\left(II\right)+yNi\left(II\right)+{2e}^{-}={\left(xCo(I)-yNi(I)\right)}_{ads}\;$$

(10) $$\;{\left(xCo(I)-yNi(I)\right)}_{ads}+e^{-}=xCo\left(s\right)+yNi\left(s\right)$$

(11) $$Cu\left(II\right)+{2e}^{-}=Cu\left(s\right)\;$$

Since the ratio of Co/Ni in the deposit is nearly ten times that of the ions in the electrolyte, an additional mechanism to describe Co(II) deposition includes the following:

(12) $$zCo\left(II\right)+e^{-}={zCo(I)}_{ads}\;$$

(13) $${zCo(I)}_{ads}+e^{-}=zCo(s)$$

where the adsorbed Co(I) species compete against the adsorption of Ni(I) species that are not coupled with Co(I), mirroring Equations (12) and (13) for Ni. Assuming the Co(I) and Co(I)-Ni(I) adsorbed species dominate, the ratio of Co/Ni in the deposit would reflect the stoichiometry (x + z)/y.

The subsequent nitrate reduction is influenced by many factors, including the current density or potential and the composition and structure of the electrocatalyst. It also competes with the H_2_ evolution reaction, in which both nitrate and hydrogen intermediates adsorb onto the electrode surface. It has been noted in the literature [48] that adsorbed H can block active sites for nitrate intermediates and block their reduction, although here, even with a relatively low FE, nitrate does react, albeit not very efficiently, and the composition can help to tune the products between ammonia and nitrogen, despite the presence of Cl^−^ that was expected to promote the nitrogen product.

Additionally, the nitrate reduction experiments presented here were conducted in a single-compartment cell, where any nitrite formed, Equation (3), can also oxidize back to nitrate. That may explain why no nitrite was detected. Also, this contributes to a lower FE that is not accounted for in Equation (8). Since Cl^−^ is present, the chlorine evolution reaction (CER) could compete with the oxygen evolution reaction (OER), where the dissolved *Cl*_2_ product would then hydrolyze to hypochlorous acid, HOCl, a strong oxidizer that could oxidize nitrite back to nitrate. However, Co-Ni-Cu alloys are known to be OER electrocatalysts [49,50], and since the OER is also more thermodynamically favored than the CER, as well as nitrite oxidation, it is unlikely that a significant amount of HOCl was generated.

Table 6 compares the nitrate conversion and products of the ternary alloy with two comparable binary alloys, electrodeposited from a similar electrolyte at −15 mA/cm^2^ for one electrolysis condition and at −5 mA/cm^2^. The ternary deposit was the same deposit as listed in Table 2. For a similar comparison, the binary alloy was electrodeposited from an electrolyte having the same copper ion concentration (0.008 M), and with the total less noble metal, either Ni or Co, having a concentration of 0.7 M, comparable to the sum of the molarities of Ni and Co in the ternary electrolyte. Table 6 shows that the alloy composition of the Cu in the binary Co-Cu and Ni-Cu alloys was similar. Following the electrolysis in the 50 mg-N/L of nitrate electrolyte containing the same sodium chloride and sodium sulfate concentrations, all three deposits, the binary and ternary, had comparable conversion of nitrate after 2 h of electrolysis. However, the ternary deposit had no nitrite detected compared to the binary alloys. Even though the ternary deposit was Co-rich, it had comparable N_2_ selectivity to the binary Ni-Cu deposit. The Co-Cu alloy had a better selectivity for ammonia but also was accompanied by a significant amount of nitrite. These results show the benefit of the ternary alloy but also underscore the complex behavior of deposit composition on nitrate reduction.

The ternary electrodeposit offers an additional compositional parameter to tune nitrate reduction products and may be of commercial and scientific interest if stability is ensured. Although stability was not part of this study, it is planned for future work. For the near-neutral-pH range investigated in these electrolysis experiments, the known overall reactions predict a local pH increase. It has been observed that copper alone will oxidize under these conditions, producing a dark copper oxide film. Such oxidation was not observed visually with the alloys presented here. To fully understand the mechanism and controlling features at the interface during nitrate reduction on ternary alloy cathodes, x-ray diffraction (XRD), x-ray photoelectron spectroscopy (XPS), and electrochemical impedance spectroscopy can be helpful but are beyond the scope of this study and are planned for future work.

## 5. Conclusions

Citrate electrolytes were designed to obtain electrodeposited Co-Ni-Cu alloy films onto a steel RCE with controlled Co/Ni ratios. The ratio of Co and Ni concentrations in the solution corresponded to a tenfold increase in the Co/Ni composition of the deposit. While reflective of anomalous codeposition, the consistency of the tenfold ratio in composition with current density points to the coupled reduction rate of Co(II) and Ni(II). The surface morphology was also governed by this ratio, with a more needle-like surface at high Co/Ni ratios and a mix of nodules and needle-like morphology at lower Co/Ni ratios. The electrodeposit with large nitrate conversions occurred when the composition ratio to deposition current density, $\frac{\frac{\left(X_{Ni}+X_{Co}\right)}{X_{Cu}}}{i},$ was between −0.51 and −0.53 cm^2^/mA, suggesting that not only composition governed nitrate reduction but that factors related to the applied current density, such as morphology, were also important. The product selectivity for nitrogen was high at high nitrate electrolysis current densities (i.e., −15 mA/cm^2^) over a range of Co-Ni-Cu composition, but the selectivity for ammonia could also be high at a low nitrate electrolysis current density (i.e., −5 mA/cm^2^) for deposits with high Cu. These findings demonstrate the rich possibilities that electrodeposited Co-Ni-Cu thin films can offer in tailoring the nitrate conversion and product selectivity.

## Figures and Tables

**Table 1:** Composition of electrodeposition electrolyte containing different amounts of Ni(II) and Co(II) with a constant Cu(II) sodium citrate and sodium lauryl sulfate concentration and 10 mL 28–30% ammonium hydroxide.

Co(II)/Ni(II)	Metal Electrolyte Composition, M				
	Ni	Co	Cu	Na_3_C_6_H_5_O_7_	NaC_12_H_25_SO_4_
1	0.35	0.35	0.008	0.25	0.00347
0.67	0.42	0.28	0.008	0.25	0.00347
0.4	0.5	0.2	0.008	0.25	0.00347

**Table 2:** Composition of the deposit after electrodeposition of electrolyte solution containing: 0.35 M Ni(II), 0.35 M Co(II), and 0.008 M Cu(II). The molar Co(II)/Ni(II) ratio in the solution is 1.

Deposition Current Density, mA/cm^2^	Metal, wt%			Thickness, μm	Co/Ni
	Ni	Co	Cu		
−5 mA/cm^2^	5.9	59.8 ± 2.7	34.3 ± 2.7	3.7 ± 0.8	10.1
−10 mA/cm^2^	6.9 ± 0.2	74.6 ± 1.2	18.5 ± 1.4	5.3 ± 0.5	10.8
−15 mA/cm^2^	7.5 ± 0.1	79.5 ± 0.4	13 ± 0.5	4.7 ± 0.7	10.6
−20 mA/cm^2^	8.3 ± 0.5	83.1 ± 0.8	8.61 ± 1.3	3.3 ± 1.1	10.0

**Table 3:** Composition of the deposit after electrodeposition of electrolyte solution containing: 0.42 M Ni(II), 0.28 M Co(II), and 0.008 M Cu(II). The molar Co(II)/Ni(II) ratio in the solution is 0.67.

Deposition Current Density, mA/cm^2^	Metal, wt%			Thickness, μm	Co/Ni
	Ni	Co	Cu		
−5 mA/cm^2^	7.3 ± 0.3	52.9 ± 1.4	39.8 ± 1.7	3.0 ± 0.3	7.2
−10 mA/cm^2^	9.9 ± 0.6	74.3 ± 1.1	15.8 ± 1.7	4.1 ± 0.3	7.5
−15 mA/cm^2^	9.9 ± 0.2	72.8 ± 0.3	17.3 ± 0.3	7.7 ± 0.8	7.4
−20 mA/cm^2^	11.4 ± 0.3	80 ± 0.3	8.6 ± 0.5	3.1 ± 0.5	7.0

**Table 4:** Composition of the deposit after electrodeposition of electrolyte solution containing: 0.5 M Ni(II), 0.2 M Co(II), and 0.008 M Cu(II). The Co(II)/Ni(II) ratio in the solution is 0.4.

Deposition Current Density, mA/cm^2^	Metal, wt%			Thickness, μm	Co/Ni
	Ni	Co	Cu		
−5 mA/cm^2^	12.7 ± 0.3	55.9 ± 1.7	31.4 ± 2.0	4.7 ± 0.2	4.4
−10 mA/cm^2^	16 ± 0.2	68.2 ± 0.8	15.8 ± 0.9	3.9 ± 0.3	4.3
−15 mA/cm^2^	16.9 ± 0.3	71.5 ± 0.9	11.6 ± 1.3	4.7 ± 0.2	4.2
−20 mA/cm^2^	18.1 ± 0.2	71.6 ± 0.3	10.3 ± 0.5	5.2 ± 0.2	4.0

**Table 5:** Composition of each best-performing deposit from Figure 4 divided by the current density.

Deposition Current Density, mA/cm^2^	Electrolyte	Metal, wt%			$\frac{\frac{\left(X_{Ni}+X_{Co}\right)}{X_{Cu}}}{i}$, $\frac{\%}{\%mA/cm^{2}}$
		Ni	Co	Cu	
−5 mA/cm^2^	(a) Co(II)/Ni(II) 1	5.9	59.8	34.3	−0.13
* −20 mA/cm^2^	(a) Co(II)/Ni(II) 1	8.3	83.1	8.6	−0.53
* −10 mA/cm^2^	(b) Co(II)/Ni(II) 0.67	9.9	74.3	15.8	−0.53
* −15 mA/cm^2^	(c) Co(II)/Ni(II) 0.4	16.9	71.5	11.6	−0.51

* indicated the highest nitrate conversion achieved for the electrolyte.

**Table 6:** Comparison of the ternary Co-Ni-Cu alloy with Co-Cu and Ni-Cu.

Electrodeposition at −15 mA/cm^2^			Nitrate Electrolysis at −5 mA/cm^2^			
Alloy	Electrolyte Solution	Cu Composition of Metals	Conversion of Nitrate	S_NH3_	S_NO2_^−^	S_N2_
Ni-Co-Cu	0.35 M Ni, 0.35 M Co, 0.008 M Cu	13 wt% Cu	24.1%	35.9%	0%	64.0%
Co-Cu	0.7 M Co, 0.008 M Cu	16.2 wt% Cu	23.6%	55.1%	44.8%	0.1%
Ni-Cu	0.7 M Ni, 0.008 Cu	14.4 wt% Cu	25.2%	23.5%	13.2%	63.3%

## Data Availability

The original contributions presented in this study are included in the article. Further inquiries can be directed to the corresponding author.
